# Assessing the Mutational Consequences of Chronic Arsenic Exposure Using Patient‐Derived Tumor Samples and In Vitro Systems

**DOI:** 10.1002/em.70079

**Published:** 2026-10-24

**Authors:** Niyati Jain, Kathryn Demanelis, Evan Wu, Muhammad G. Kibriya, Farzana Jasmine, Emma Wilkinson, Syed Emdadul Haque, Mohammed Kamal, Saequa Habib, Qamruzzaman Chowdhury, Farida Arjuman, Habibul Ahsan, Lixing Yang, Yu‐Ying He, Brandon L. Pierce

**Affiliations:** ^1^ Department of Public Health Sciences University of Chicago Chicago Illinois USA; ^2^ Committee on Genetics, Genomics, Systems Biology University of Chicago Chicago Illinois USA; ^3^ Department of Medicine University of Pittsburgh Pittsburgh Pennsylvania USA; ^4^ UPMC Hillman Cancer Center Pittsburgh Pennsylvania USA; ^5^ Center for Research Informatics Bioinformatics Core University of Chicago Chicago Illinois USA; ^6^ Institute for Population and Precision Health (IPPH), Biological Sciences Division University of Chicago Chicago Illinois USA; ^7^ Section of Dermatology, Department of Medicine University of Chicago Chicago Illinois USA; ^8^ UChicago Research Bangladesh Dhaka Bangladesh; ^9^ Bangladesh Medical University (BMU) Dhaka Bangladesh; ^10^ Ahsania Mission Cancer & General Hospital Dhaka Bangladesh; ^11^ National Institute of Cancer Research and Hospital (NICRH) Dhaka Bangladesh; ^12^ Ben May Department for Cancer Research University of Chicago Chicago Illinois USA; ^13^ Department of Human Genetics University of Chicago Chicago Illinois USA; ^14^ Comprehensive Cancer Center University of Chicago Chicago Illinois USA

**Keywords:** arsenic, cancer, environment, mutational signature, somatic mutations

## Abstract

Inorganic arsenic, a naturally occurring metalloid found in groundwater, is a well‐established human carcinogen. Epidemiological studies from highly contaminated regions link chronic arsenic exposure to increased risks of skin, lung, bladder, and kidney cancer. However, studies investigating the mechanisms driving arsenic‐related mutagenicity in human tumors remain limited. To improve our understanding, we analyzed whole‐exome sequencing (WES) data from tumor samples of 17 cancer patients treated in Dhaka, Bangladesh, with known or suspected exposure based on toenail arsenic concentrations. These patients included bladder (*n* = 9), kidney (*n* = 6), and lung (*n* = 2) cancers. The mutational burden (mutations per megabase) across all exomes ranged from 23 to 117 for single‐base substitutions (SBS) and 2 to 13 for small insertions and deletions (indel). We showed that SBS and indel signatures, including SBS5, SBS6, ID6, and ID19 contributed the highest mutation load across all exomes. However, we did not observe associations between arsenic exposure levels and any mutational signature. We additionally analyzed WES data from two cell line models of chronic arsenic exposure, HaCaT skin epithelial and BEAS‐2B lung epithelial cells exposed to low‐dose of arsenite (100 nM) for 28 weeks. Consistent with the patient‐derived results, we observed no exposure‐related mutational burden or signature. Taken together, our findings suggest that arsenic alone may have limited independent mutagenic effects and instead may act through co‐carcinogenic mechanisms. However, limited sensitivity to detect low‐frequency mutations and small sample sizes potentially reduced our ability to capture mutational processes that may be associated with chronic arsenic exposure.

## 1 Introduction

Chronic exposure to inorganic arsenic through consumption of contaminated water and food is a major public health issue that affects > 230 million people worldwide. In Bangladesh, > 50 million people are exposed to arsenic through drinking water tube‐wells, the primary source of exposure (Ahmad et al. 2018; Shaji et al. 2021). Arsenic exposure is associated with increased risk of skin lesions and several types of cancer including bladder, liver, kidney, skin, and lung cancer (Martinez et al. 2011). Arsenic is classified as a Group I human carcinogen by the International Agency for Research on Cancer (IARC), but the molecular mechanisms underlying its carcinogenic effects remain poorly understood. Unlike other environmental carcinogens such as tobacco smoke (Alexandrov et al. 2016) and ultraviolet (UV) radiation (Alexandrov et al. 2020), arsenic has not yet been associated with a clear, reproducible mutational signature. Mutational signatures are a characteristic pattern of single base pair substitutions (SBS), small insertion–deletion (indel), dinucleotide (DBS), structural variant (SV), or copy number variant (CNV) mutations that accumulate in response to environmental and endogenous mutagens (Islam et al. 2022).

Early in vitro studies in human‐hamster hybrid (A_L_) cells showed that arsenite (trivalent form of inorganic arsenic) is a potent mutagen whose activity is at least partly dependent on the presence of reactive oxygen species to induce large deletion mutations and chromosomal abnormalities (Hei et al. 1998; Kessel et al. 2002; Liu et al. 2001). Similarly, a study of a human lung fibroblast cell line reported that arsenite induces single‐strand breaks and protein‐DNA adducts in exposed cells (Mourón et al. 2006). Exposure to arsenite in HaCaT human skin epithelial cells has been shown to induce oxidative stress and inhibit a key DNA excision repair protein, poly(ADP‐ribose) polymerase‐1 (PARP‐1) (Qin et al. 2008). Other experimental studies have proposed epigenetic and epitranscriptomic mechanisms underlying arsenic‐induced carcinogenesis. For example, arsenic exposure has been associated with alterations in global histone methylation patterns (Zhou et al. 2008), aberrant DNA methylation of a tumor suppressor gene (Mass and Wang 1997), and changes in N6‐methyladenosine (m6A) RNA demethylase activity (Cui et al. 2021; Wilkinson et al. 2025). Additionally, dysregulation of microRNA expression has been reported as a potential contributing mechanism driving arsenic‐related tumorigenesis (Cui et al. 2012). A human observational study examining the chromosomal alterations in bladder tumors from 123 patients from Chile observed that the number of chromosomal alterations was associated with levels of arsenic exposure (Moore et al. 2002). Together, these findings suggest that arsenic exposure is capable of inducing DNA damage and genomic instability, but they do not address whether arsenic leaves a distinct mutational signature.

Recent efforts to characterize arsenic‐related mutational patterns using sequencing approaches have produced conflicting results. One study reported the first whole‐genome sequence of a lung squamous cell carcinoma (LUSC) from a never‐smoker chronically exposed to arsenic and described a distinct mutational spectrum, including an elevated fraction of T>G/A>C mutations and a G>C mutation in tumor suppressor protein p53 (TP53) (Martinez et al. 2013), which is uncommon in LUSC but has been observed in other arsenic‐related tumors (Hsu et al. 1999; Shibata et al. 1994). However, the LUSC study was limited to a single patient. In vitro whole‐genome sequencing (WGS) experiments, by contrast, found that short‐term exposure of cells—immortalized non‐cancerous human keratinocyte cell line (N/TERT1) (Speer, Nandi, et al. 2023) and human induced pluripotent cells (iPSCs) (Kucab et al. 2019)—to high concentrations of arsenite alone does not result in an increased mutational burden or a distinct mutational signature. This study proposed that arsenic may act as a co‐mutagen and co‐carcinogen, enhancing other mutational processes, such as those related to UV radiation (Speer, Nandi, et al. 2023). However, these in vitro studies are limited in scope as they examined short‐term exposure (24–48 h) to high concentrations of arsenic, which is not representative of typical human exposure associated with skin lesions and cancer. Experimental studies of chronic, low‐level arsenic exposure have demonstrated malignant transformation in various human and rodent cells (Pi et al. 2008; Zhao et al. 1997; Achanzar et al. 2002; Chien et al. 2004; Wen et al. 2008), making it a better model to assess arsenic's mutational impact.

In this work, we analyzed whole‐exome sequencing (WES) data from 17 Bangladeshi patients with varying levels of arsenic exposure and diagnosed cancers of arsenic‐relevant organs (lung, bladder, and kidney). We also examine WES data from experimental model systems chronically exposed to low‐dose arsenite, including an immortalized, human adult keratinocyte cell line (HaCaT) (Cui et al. 2021) and *a normal human bronchial epithelium* cell line (BEAS‐2B) (Wilkinson et al. 2025), as previously described. By studying both population‐based human tumor data and controlled in vitro systems that better reflect real‐world exposure, we aimed to systematically investigate the mutagenic impact of arsenic and clarify whether arsenic leaves a reproducible mutational signature that contributes to its carcinogenesis mechanism.

## 2 Methods

### 2.1 Study Population

This cross‐sectional study was conducted across five major medical institutions in Dhaka, Bangladesh. We recruited 76 patients with histologically confirmed tumors in arsenic‐relevant organs including lung, kidney, bladder, and skin. Patients' formalin‐fixed paraffin‐embedded (FFPE) tumor tissue blocks were obtained from: Bangladesh Medical University (BMU), the National Institute of Cancer Research & Hospital (NICRH), the National Institute of Diseases of the Chest and Hospital (NIDCH), Ahsania Mission Cancer and General Hospital (AMCGH) and a private histopathology laboratory, The Laboratory. Patients whose tumor blocks were obtained from The Laboratory had undergone surgery at different medical facilities in Dhaka, such as BMU, NICRH, and AMCGH.

After obtaining informed consent for study participation, including permission to use tumor tissue blocks, study physicians conducted in‐person interviews using a standardized questionnaire to collect demographic and lifestyle information including age, sex, smoking status, occupation, residential district, and education level. At the time of interview, toenail clippings were collected with the assistance of laboratory personnel for exposure assessment. Data confidentiality was maintained using unique patient IDs. Laboratory and data management support were provided by UChicago Research Bangladesh.

### 2.2 Elemental Analysis of Toenail Samples

Toenail clippings were stored in metal‐free plastic bags and shipped to Chicago, Illinois, United States. Samples were processed at the Mass Spectrometry Core in the Research Resources Center of the University of Illinois at Chicago, supported by The ChicAgo Center for Health and EnvironmenT (CACHET), following standard preparation procedures. The nail samples were cleaned, acid washed, freeze‐dried, and weighed prior to analysis by the Perkin Elmer NexION 2000 inductively coupled plasma mass spectrometry (ICP‐MS) with the standard 75 mL Teflon digestion vessels. The standard curve range suite included aluminum, vanadium, chromium, iron, manganese, cobalt, nickel, copper, zinc, molybdenum, cadmium, tin, lead, arsenic, selenium, and antimony.

### 2.3 Cell Culture

We used previously generated HaCaT and BEAS‐2B cells, including cells continuously treated with 100 nM of inorganic arsenite (sodium arsenite; Millipore Sigma Cat# 1062771000) for 28 weeks to generate cells with chronic arsenic damage (As‐treated), and non‐treated control (NT) cells passage‐matched to As‐treated cells, described in Cui et al. (2021) and Wilkinson et al. (2025), respectively. The 100 nM concentration was selected because it is a physiologically relevant dose and comparable to blood arsenic levels reported in individuals with chronic arsenosis (Pi et al. 2000; Y.‐H. Cui et al. 2021; Wilkinson et al. 2025; Kim et al. 2021; Pi et al. 2008). A 28‐week exposure period was used to model chronic, low‐level exposure, consistent with established in vitro models of arsenic‐induced malignant transformation (Pi et al. 2000; Cui et al. 2021; Wilkinson et al. 2025; Kim et al. 2021; Pi et al. 2008). HaCaT (kindly provided by Dr. Norbert Fusenig) and BEAS‐2B (ATCC, CRL‐3588; RRID: CVCL_0168) cells were maintained in Dulbecco's modified Eagle's medium (Thermo Fisher Scientific, 11965118), supplemented with 10% fetal bovine serum (Gibco, 10–437‐028), 100 U/mL penicillin, and 100 μg/mL streptomycin (Invitrogen, 15140122). For As‐treated cells, cells were continuously maintained in medium (DMEM) containing 100 nM of sodium arsenite (Wilkinson et al. 2025; Cui et al. 2021). We performed independent DNA extractions from the same As‐treated or NT cell population, generating five technical replicates per condition. Parent stock cell line was used as the matched normal for somatic mutation calling.

### 2.4 Whole Exome Sequencing

DNA was extracted from FFPE human tissue samples using the Quick‐DNA FFPE Kit, Zymo Research Catalog Nos. D3067, following the manufacturer's protocol. For cell culture samples, DNA was extracted using the QIAamp DNA Mini Kit (Qiagen) following the manufacturer's protocol. DNA quantification was checked by Qubit fluorometer. DNA libraries and exome target enrichment for sequencing were prepared using the Twist Bioscience Library Preparation Enzymatic Fragmentation Kit 2.0 and Twist Bioscience Exome 2.0 following the manufacturer's protocol. Qualified libraries were sequenced using Illumina NovaSeqX. Notably, of the 76 tumor blocks collected, only 66 yielded DNA of sufficient quality for sequencing. No exclusions were made for the cell culture samples.

### 2.5 Quality‐Control and Detection of Somatic Mutations From Whole‐Exome Sequencing Data

We applied the open‐source workflow, Sarek version 3.4.2 (Garcia et al. 2020), to perform quality‐control (QC) and detect somatic mutations using the WES data. Quality of raw FASTQ files was evaluated using FASTQC and FASTP. Raw sequencing reads were aligned to the human reference genome (GRCh38). The aligned reads were marked duplicates using GATK MARKDUPLICATES, and coverage was computed using Mosdepth (Pedersen and Quinlan 2018). Finally, GATK Mutect2 was used for SNV and indel somatic mutation calling.

Mutect2 uses a Bayesian somatic genotyping model and novel filtering scheme that can handle samples with and without a matched normal and with varying sequencing depths to detect somatic mutations with confidence (Benjamin et al. 2019). Mutation calling was restricted to exome intervals specified in Twist Bioscience's Exome 2.0 interval bed file (https://www.twistbioscience.com/resources/data‐files/twist‐exome‐20‐bed‐files). We included 100 base pair (bp) padding to account for sequencing reads that extended outside of exome intervals. FFPE‐preserved human tumor samples lacking matched normal tissue were analyzed using Mutect2's “*tumor‐only*” mode. We specified the default GRCh38 iGenomes configuration (https://github.com/nf‐core/sarek/blob/master/conf/igenomes.config) within Sarek, which provides the human reference genome, panel of normals (pon) from the 1000 Genomes Project, and the genome aggregate database (gnomAD) as the germline resource. Somatic mutations are detected based on pon evidence, population allele frequencies, and variant allele frequencies within the tumor sample. For cell line samples, we implemented Mutect2's “tumor with matched normal” mode which additionally filters calls based on presence in the matched normal sample. Because FFPE samples can exhibit read‐orientation artifacts, we used the “LearnReadOrientationModel” in Mutect2 to generate orientation‐bias artifact priors, which were then incorporated into “FilterMutectCalls” to filter variants with evidence of orientation bias. Final mutation calls were selected based on Mutect2's default filtering criteria (i.e., variants labeled “PASS”). For cell lines, we further excluded mutations detected in both As‐treated and NT conditions, as these likely represented pre‐existing variants not captured in the sequencing of the parental stock rather than condition‐specific somatic events. Mutational matrices for SBS, DBS, and indel mutations were generated with SigProfilerMatrixGenerator (Bergstrom et al. 2019).

For the 66 human tumor WES samples, we applied a minimum coverage threshold of 30× for sample inclusion. This threshold was selected to balance data quality, while maximizing sample size, since using a more stringent threshold (e.g., 100×) would have substantially reduced the number of samples available for analysis (Figure S1). We further restricted to tissue types with multiple samples available (lung, kidney, and bladder), excluding individuals diagnosed with benign tumors and with no corresponding toenail arsenic measurements. Following these filtering steps, 17 malignant tumor samples were retained for downstream analysis. For the cell culture samples, no exclusions were made based on coverage, as minimum coverage across samples exceeded 100×.

### 2.6 Analysis of Mutational Signatures

Analysis of mutational signatures was performed using the SigProfiler suite of tools for simulation, assignment, and visualization of mutational signatures. De novo mutational signature extraction and COSMIC decomposition of de novo signatures were performed with SigProfilerExtractor (Islam et al. 2022). Attribution of COSMIC signatures (version 3.4, https://cancer.sanger.ac.uk/signatures/) (Alexandrov et al. 2020, Forbes et al. 2017)—a catalog of curated reference mutational signatures—to individual mutational profiles was performed using SigProfilerAssignment (Díaz‐Gay et al. 2023). SigProfilerPlotting was used to visualize mutational profiles of the cancer exomes (Khandekar et al. 2023).

## 3 Results

### 3.1 Overview of Data Sources and Analysis Pipeline

We analyzed tumor samples from 17 Bangladeshi patients with diagnosed cancers in arsenic‐relevant tissues,including bladder (*n* = 9), kidney (*n* = 6), and lung (*n* = 2) as well as toenail arsenic concentrations, which reflect exposure from the previous 5–18 months (Signes‐Pastor et al. 2021). Histopathological review confirmed cancer subtypes, including bladder urothelial cancer (*n* = 9), kidney urothelial cancer (*n* = 3), papillary renal cell carcinoma (*n* = 2), sarcomatoid renal cell carcinoma (*n* = 1), lung adenocarcinoma (*n* = 1), and small cell lung carcinoma (*n* = 1). Patient ages ranged from 21 to 70 years, with 71.6% of individuals self reported as male and 56% classified as never smokers (Table 1).

**TABLE 1:** Characteristics of cancer patients from Bangladesh.

Cancer site	Bladder (*n* = 9)	Kidney (*n* = 6)	Lung (*n* = 2)	All (*n* = 17)
Subtypes (*n*)	Urothelial (9)	Urothelial (3)	Adenocarcinoma (1)	—
		Papillary RCC (2)	Small cell carcinoma (1)	
		Sarcomatoid RCC (1)		
Age, years (SD)	53.8 (12.8)	37.8 (11.2)	57.5 (3.5)	48.6 (13.8)
Gender (%)				
Female	3 (33)	1 (16.7)	1 (50)	5 (29.4)
Male	6 (67)	5 (83.3)	1 (50)	12 (71.6)
Smoking (%)				
Ever	4 (44.5)	2 (33)	1 (50)	7 (42)
Never	5 (55.5)	4 (67)	1 (50)	10 (56)

We utilized previously proven in vitro systems for studying effects of long‐term arsenic exposure; specifically, HaCaT cells (Cui et al. 2021), a human keratinocyte cell line, and BEAS‐2B cells (Wilkinson et al. 2025), a human lung epithelial cell line, were continuously treated with a physiologically relevant dose (100 nM) of inorganic arsenite for 28 weeks to generate cells with chronic arsenic damage (As‐treated) cells, while non‐treated (NT) control cells were passage‐matched to As‐treated cells. These cell lines were selected as they are derived from arsenic‐relevant organs (skin and lung) and are immortalized lines but non‐tumorigenic, making them well suited for investigating the molecular mechanisms underlying arsenic‐induced malignant transformation (Cui et al. 2021; Kim et al. 2021; Wilkinson et al. 2025). Tumor (Figure 1A) and cell line samples (Figure 1B) were subjected to WES, followed by mutational burden and mutational signature analysis.

**FIGURE 1:**
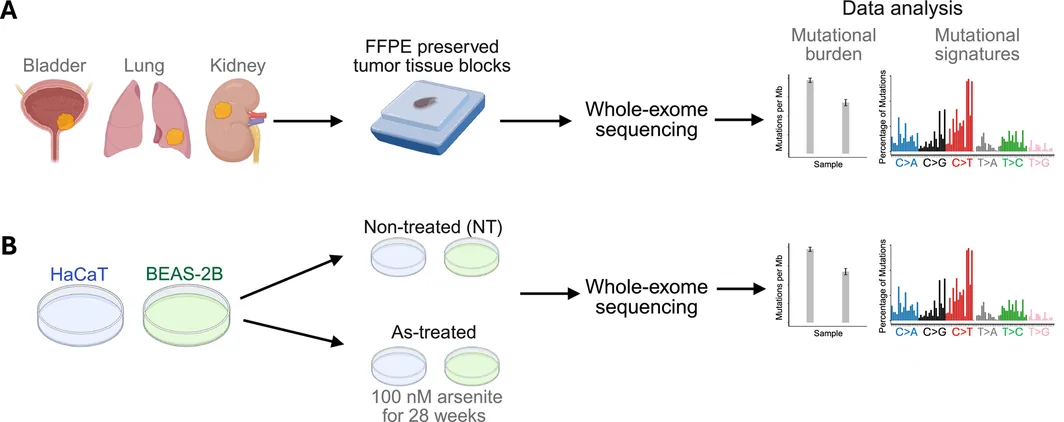
Overview of data sources and analysis. (A) human tumor samples (B) cell line models, including HaCaT skin epithelial and BEAS‐2B lung epithelial cell line. *Source:* Created in BioRender. Jain, N. (2027) https://BioRender.com/djdkuh1.

### 3.2 Arsenic Exposure Levels Among Bangladesh Patient Cohort

At the time of recruitment, all patients who provided district‐level information (*n* = 51) reported they resided outside the capital city Dhaka and had traveled to the city to receive treatment. Using district‐level arsenic data from Chakraborti et al. 2010, who collected 52,202 tubewell water samples across all 64 districts of Bangladesh, we observed substantial variation in the proportion of tubewells exceeding 10 μg/L across the regions represented in our patient cohort (Figure S2) (Chakraborti et al. 2010).

Toenail arsenic concentrations among the subset of participants included in our analysis (*n* = 17) ranged from 0.09 μg/g to 19.7 μg/g; aside from two individuals with highly elevated levels (> 10 μg/g), all remaining individuals had concentrations below 2.3 μg/g. The mean and median arsenic concentration were 2.6 and 0.4 μg/g, respectively (Figure 2A and Figure S3). These exposure levels are comparable to those reported by a prior meta‐analysis that analyzed several studies in exposed populations from South Asia, including India and Bangladesh, and are expectedly higher than levels reported in populations living in the United States, Australia, and Canada, where mean and median concentrations were reported to be < 0.05 μg/g (Signes‐Pastor et al. 2021).

**FIGURE 2:**
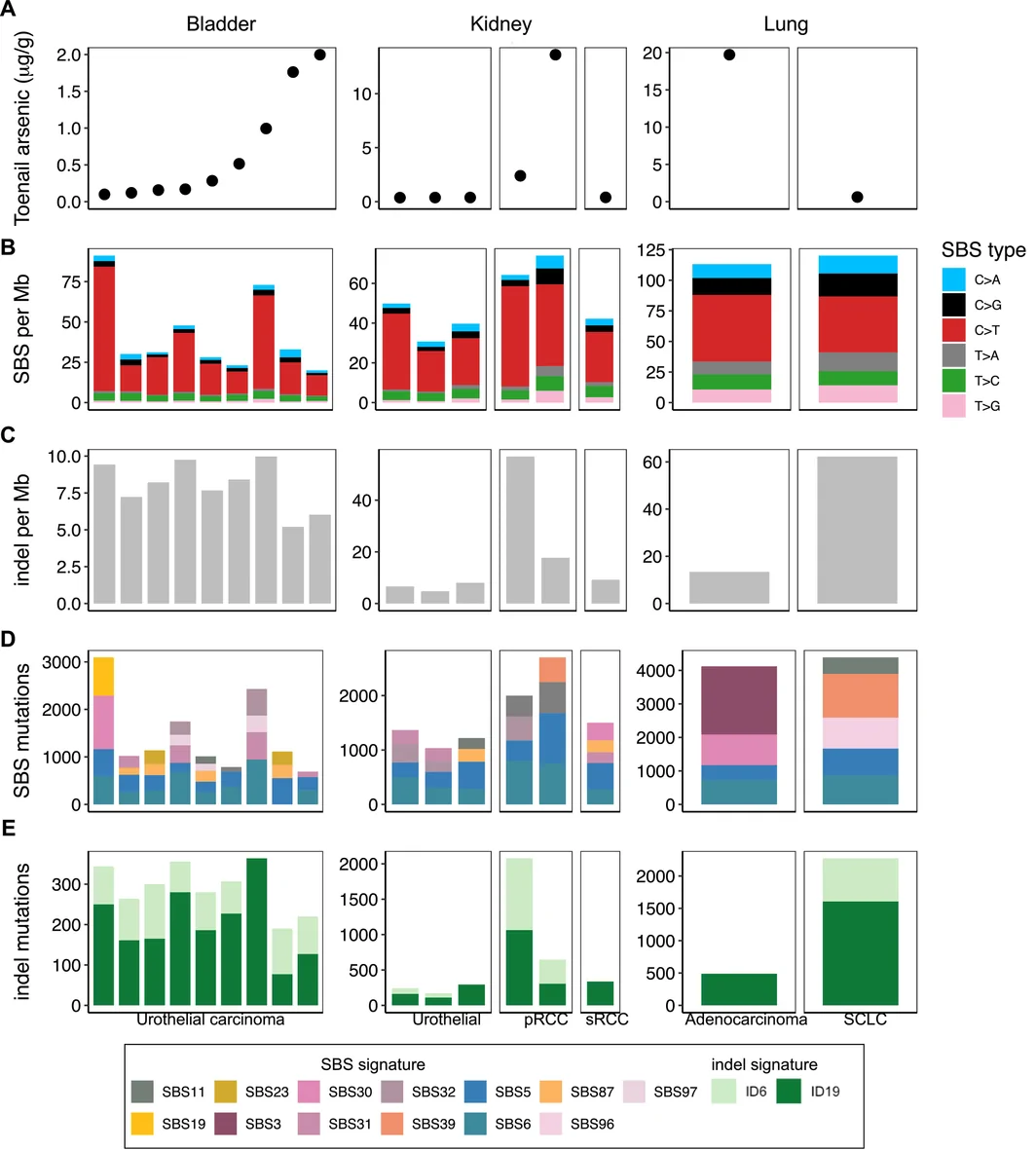
No clear link between mutational burden or mutational signatures and arsenic exposure measured in toenails (A) Toenail arsenic concentrations per sample. (B) Number of single‐base substitutions (SBS) per megabase (Mb) per sample. (C) Number of small insertions and deletions (indels) per Mb per sample. (D) Number of mutations attributed to each COSMIC SBS mutational signature. (E) Number of mutations attributed to each COSMIC indel mutational signature. Mutational signatures contributing less than 10% of mutations to the total mutational profile are not shown. For each cancer subtype, the ordering of bars in panels B–D is based on sample arsenic concentrations shown in panel A. pRCC, papillary renal cell carcinoma; SCLC, small cell lung carcinoma; sRCC, sarcomatoid renal cell carcinoma.

### 3.3 Examining the Mutational Profiles of Human Tumors

To assess the relationship between arsenic exposure and mutational patterns in human tumors, we applied a bioinformatic pipeline to identify somatic mutations in tumor‐only samples, followed by analyses comparing mutational burden and mutational signatures across individuals with varying levels of arsenic exposure.

We first compared the total number of SBS and indel mutations across all samples, stratified by cancer site and subtype (Figure 2). Given that our tumor samples were preserved as FFPE tissue, we accounted for mutational patterns associated with FFPE treatment by applying FFPEsig (Guo et al. 2022). After accounting for such artifacts—defined predominantly by C>T substitutions—C>T remained the most frequent SBS across all cancers. Prior studies have described that C>T mutations at methylated CpG sites arising from the deamination of 5‐methylcystosine are prevalent in both normal and cancer cells and may result from both biologic and technical processes, such as FFPE treatment (Guo et al. 2022; Alexandrov et al. 2015; Steiert et al. 2023).

To further assess the robustness of our findings to potential residual FFPE artifacts, we performed a sensitivity analysis using more stringent variant‐level filters as FFPE‐associated artifacts are often characterized by low allele frequencies and lower variant quality metrics (Guo et al. 2022; Do and Dobrovic 2015; Costello et al. 2013; Yost et al. 2012). We applied a minimum VAF threshold of 5% and a minimum read depth of 25× (Luo et al. 2021), followed by FFPEsig. These additional filters removed a substantial number of substitutions (563 mutations on average) across samples (Figure S4B,C), particularly C>T substitutions (Figure S5), consistent with a potential contribution of FFPE‐associated artifacts. Nevertheless, C>T remained the most frequent substitution type, and the overall SBS patterns and mutational signature profiles were comparable with and without the additional filters, suggesting that our findings are not primarily driven by technical artifacts (Figure S4C).

For indel mutations, we observed that 5 bp insertions at repetitive sequences and microhomology‐mediated deletions were the most common alterations across all cancers. The bladder urothelial cancer samples, which represent the largest subset of a single cancer type, were further analyzed using ANNOVAR (Wang et al. 2010) by functionally annotating mutations overlapping known driver genes (Vogelstein et al. 2013) (Tables S1 and S2). Notably, we did not observe a clear link between arsenic exposure levels and SBS or indel mutational burden across all cancers (Figure 2B,C).

Given the small sample sizes for cancer subtypes, we had limited statistical power to reliably extract mutational signatures de novo. Therefore, we applied SigProfiler Assignment, a numerical optimization approach that estimates the contribution of previously described COSMIC signatures (Tate et al. 2019) to individual exomes independently. This approach enabled us to evaluate whether known mutational patterns were associated with arsenic exposure levels. We found that SBS5, a clock‐like signature with no known etiology, is present at high proportions in almost all cancer samples. We additionally observed a high proportion of the SBS6 signature across all cancer samples, a signature arising from defective DNA mismatch repair and characterized by C>T mutations (Alexandrov et al. 2013). While most signatures were present in several cancer exomes, we did identify several signatures unique to individual samples, such as SBS23 (no known etiology) present in a urothelial bladder carcinoma sample, and SBS3 (defective homologous recombination‐based repair) present in the lung adenocarcinoma sample. Across all cancer samples, only two indel signatures were assigned, including ID6, associated with defective homologous recombination DNA damage repair, and ID19, characterized by 5 bp insertions with no known etiology. Finally, we did not observe a clear arsenic‐related SBS or indel mutational signature (Figure 2D,E).

Next, we assessed whether overall element burden revealed any noticeable patterns with total burden or signature profiles. We observed one individual diagnosed with kidney pRCC and one with lung adenocarcinoma who exhibited high concentrations of most elements measured (Figure S6A). These two patients were among those with the highest SBS burden (Figure S6B) but displayed low levels of indel burden (Figure S6C). We again observed no clear relationship between overall elemental burden and mutational signature profiles across all cancer exomes (Figure S6D,E).

### 3.4 Assessing Arsenic's Mutational Impact in In Vitro Systems

We leveraged in vitro systems to dissect the mutational impact of chronic arsenic exposure. Somatic mutations were identified from whole‐exome sequenced HaCaT and BEAS‐2B As‐treated cells and NT cells, followed by de novo mutational signature extraction. In HaCaT skin epithelial cells, the mutational patterns of As‐treated cells were nearly identical to those of NT cells (Figure 3A–C; left). In addition, chronic arsenic exposure alone did not result in increased numbers of SBS, DBS, or indel mutations compared to NT cells (Figure 3A–C right; Figure S7A–C). Consistent with observations in human tumor data, SBS5 contributed at high proportions across both As and NT cells. We also identified ID19 as the predominant indel signature in HaCaT cells.

**FIGURE 3:**
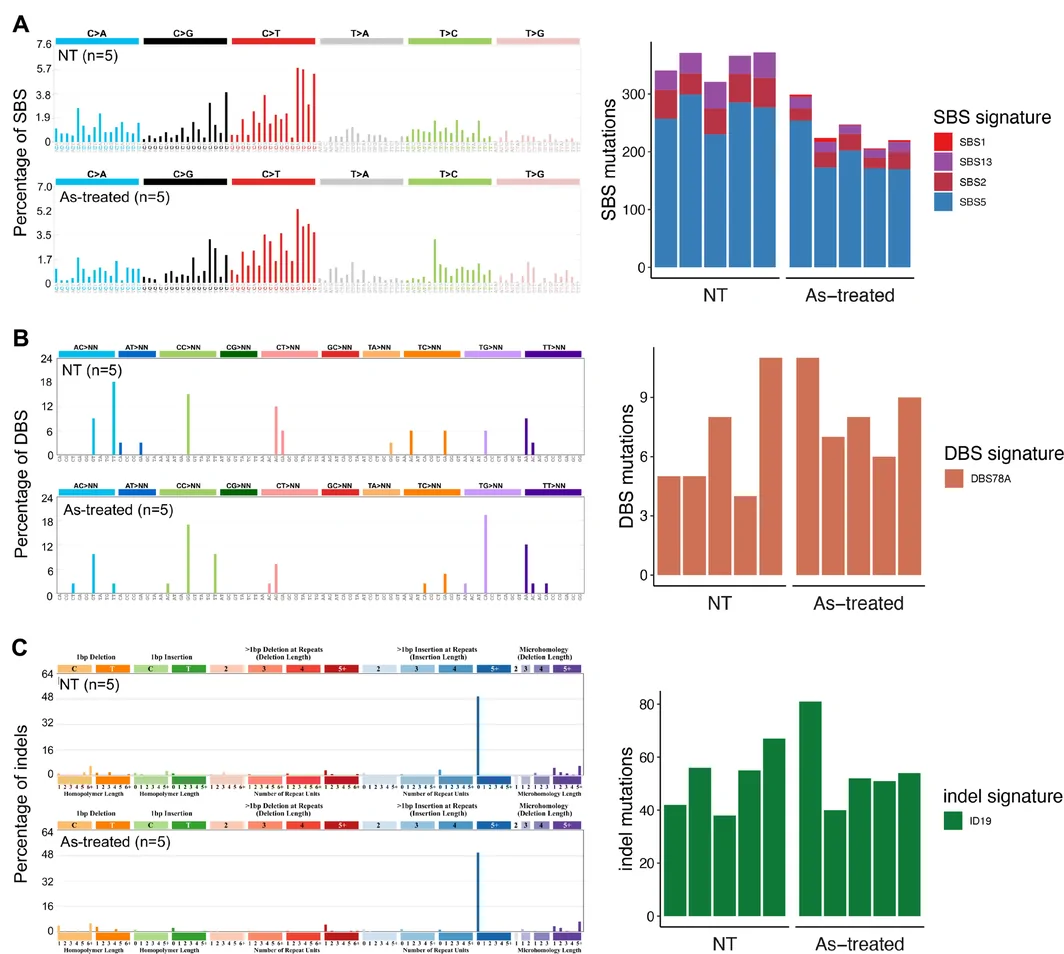
Chronic arsenic exposure does not show a clear impact on mutational burden or mutational signature in HaCaT skin epithelial cells. (A) Single base substitutions (SBS), (B) double base substitutions (DBS), and (C) small insertions and deletions (indels). For each mutation class, the average mutational pattern using the SBS96, DBS78, and ID83 COSMIC classification schemes, respectively, is shown on the left. The number of mutations attributed to each COSMIC mutational signature is shown on the right.

Consistent with HaCaT cells, the BEAS‐2B lung epithelial cell line also exhibited highly similar SBS and indel mutational profiles between arsenic‐exposed and NT conditions, with no significant differences in overall mutational burden across conditions (Figure 4A,B; Figure S8A,B). SBS18 emerged as the dominant SBS signature, and DBS mutations were not identified in this cell line. Although one arsenic‐exposed sample showed a relatively elevated indel burden, this increase was not accompanied by distinct changes in mutational signature composition (Figure 4B). Together, these findings suggest that chronic arsenic exposure alone does not substantially alter mutational burden or signature profiles in these cellular systems.

**FIGURE 4:**
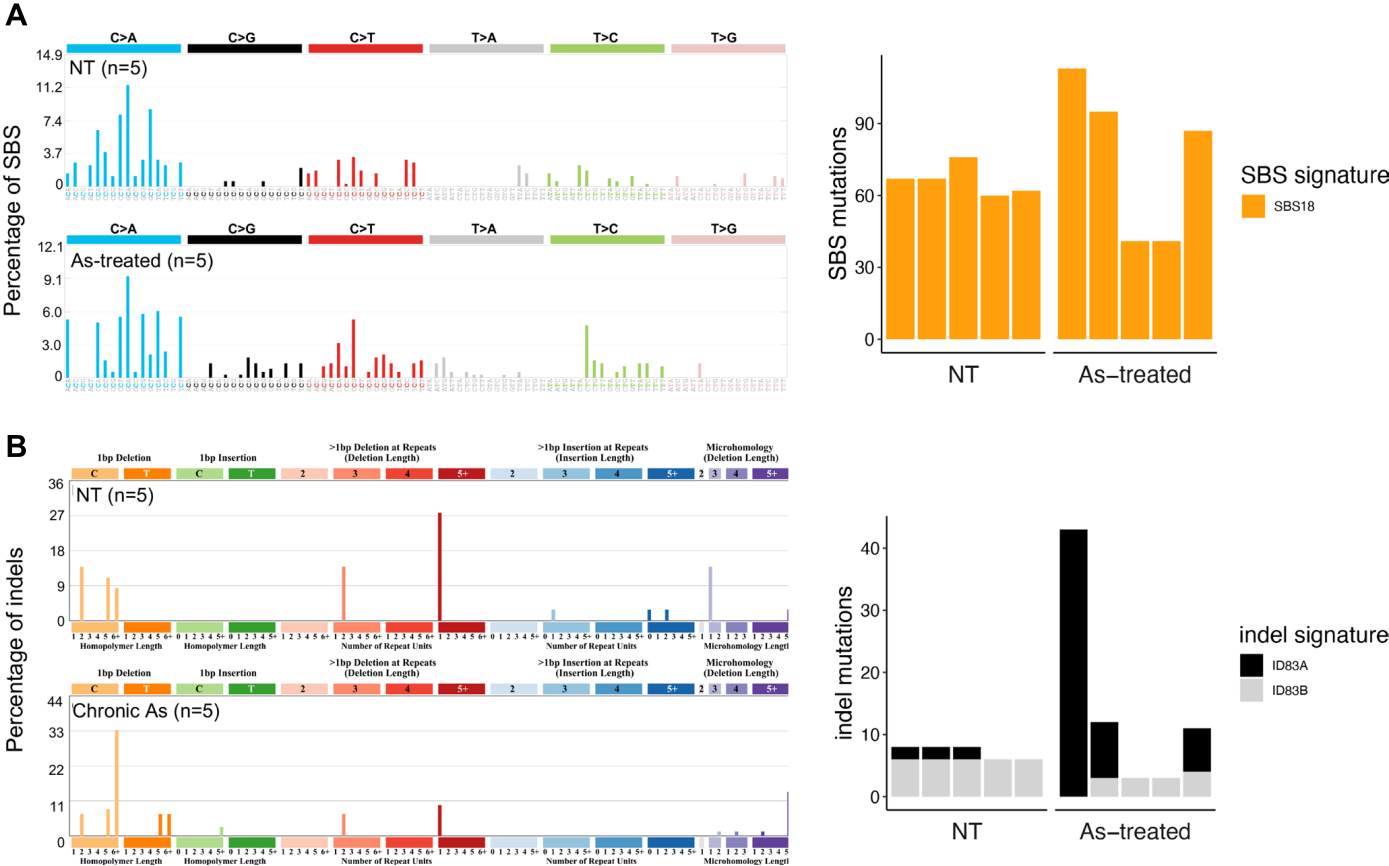
BEAS‐2B lung epithelial cells similarly lack arsenic‐associated mutational burden or mutational signature following chronic exposure (A) Single base substitutions (SBS), (B) small insertions and deletions (indels). For each mutation class, the average mutational pattern using the SBS96 and ID83 COSMIC classification schemes, respectively, is shown on the left. The number of mutations attributed to each COSMIC mutational signature is shown on the right.

## 4 Discussion

In this study, we evaluated the mutational profiles of 17 human tumors alongside two cell lines of chronic arsenic exposure. Across both the patient‐derived tumor samples and arsenic‐exposed cell line WES data, we did not observe a clear association between arsenic and an increased overall mutational burden, or a distinct or exposure‐associated signature. Our findings suggest that arsenic alone may have limited independent mutagenic or DNA‐damaging effects. One possible explanation is that arsenic contributes to cancer through co‐carcinogenesis involving a combination of mechanisms, including disruption of DNA repair pathways, generation of reactive oxygen species, and epigenetic dysregulation (Cui et al. 2021; Wilkinson et al. 2025; Speer, Zhou, et al. 2023; Zhou et al. 2021), without producing a characteristic somatic mutation profile.

Among bladder, kidney, and lung tumor samples, SBS signatures SBS5 and SBS6, and indel signatures ID6 and ID19, accounted for the largest proportion of observed mutations. However, none of these signatures demonstrated a noticeable pattern with arsenic exposure levels. SBS5 is a clock‐like signature broadly observed in normal cells and across cancer types (Alexandrov et al. 2013). Notably, a recent study in humans (Liu et al. 2024) and *Escherichia coli* (Ba et al. 2022) reported that SBS5 is a prominent signature after heavy metal exposure, although the mechanisms driving this association remain unclear and warrant further investigation. SBS6 and ID6 are associated with defective DNA damage repair, while ID19 has no known etiology. In our analysis, the lack of a clear relationship between any SBS or indel signature and arsenic exposure may reflect limited statistical power (due to small size) to detect such associations or suggest that arsenic exposure is not linked to a detectable mutational profile.

Several limitations should be considered when interpreting our findings. First, the tumor samples analyzed lacked matched normal tissue, which limits the accuracy of somatic variant calling. In addition, all samples were preserved as FFPE tissue, a method known to introduce characteristic sequencing artifacts, including C>T mutations. Such artifacts were accounted for in our somatic mutation calling pipeline and by applying FFPEsig (Guo et al. 2022). However, since FFPEsig was developed using WGS data, it is expected to not perform as well on WES data, as the signal (total mutational burden) is lower than in WGS. As such, the persistence of C>T substitutions in our samples after correction likely reflects a combination of endogenous mutational processes and residual technical bias. However, our sensitivity analysis, which applied additional VAF and read depth filters, suggested that technical artifacts were likely not the primary driver of the overall mutational patterns or signature profiles. Furthermore, a large subset of samples exhibited WES coverage below 100×, which increases uncertainty in calling somatic mutations. Although we applied established bioinformatic pipelines designed to minimize false‐positive calls, we cannot fully exclude technical effects that may influence mutation detection and signature attribution.

Additionally, while toenails are a well‐established biomarker of arsenic exposure from the previous 5–18 months, it may not be representative of exposure levels during earlier stages of carcinogenesis, when the somatic mutations observed in tumors may have been established. Furthermore, our tumor cohort was small (*n* = 17) and consisted predominantly of individuals with relatively low arsenic exposure levels, limiting our ability to evaluate the relationships between the degree of exposure and somatic mutation patterns. Future studies in larger cohorts spanning a broader range of arsenic exposure levels and integrating residential, occupational, and dietary exposure assessments may provide a more comprehensive characterization of historical arsenic exposure, enabling a better understanding of dose‐dependent mutational effects of arsenic in human tumors. Beyond technical limitations, our work highlights the logistical challenges of coordinating an international, multi‐institutional study, including patient recruitment and standardization of protocols for tumor block collection, which can affect sample size and data quality.

Consistent with the human tumor data, the in vitro models of chronic arsenic exposure did not reveal a characteristic mutational imprint or increased mutational burden associated with chronic arsenic exposure. Recent studies point to epigenetic mechanisms as playing a critical role in arsenic‐mediated tumorigenesis. Previous work has proposed that HaCaT and BEAS‐2B cells with chronic arsenic damage acquire tumorigenic properties through epitranscriptomic mechanisms involving regulation of RNA demethylases FTO and ALKBH1 (Y.‐H. Cui et al. 2021; Wilkinson et al. 2025). Another study showed that co‐exposure of oral epithelial cells to arsenic and tobacco results in upregulation of genes involved in cell cycle regulation, apoptosis, and inflammatory responses accompanied by global DNA hypomethylation of genes involved in these same processes (Das et al. 2025). Our results further raise the possibility that such mechanisms may contribute to arsenic‐driven tumorigenesis, as we find that arsenic alone does not exhibit strong independent mutagenic properties in these cellular systems.

However, limitations of the in vitro systems analyzed here should also be acknowledged. Mutational profiling was performed using bulk sequencing data of a mixed population of cells, which prevents investigation of mutational patterns at the level of individual clones. As a result, rare or subclonal mutational events potentially associated with arsenic exposure may not be captured. Future studies incorporating single‐cell sequencing or clonal expansion followed by sequencing could provide greater sensitivity to detect such rare mutational processes (Funnell et al. 2022; Zhivagui et al. 2017; Huang and Lee 2022).

Both the patient‐derived tumor analyses and in vitro experiments were limited to WES, which captures only a small fraction of the genome. A prior comparison of matched WES and WGS samples demonstrated that the majority of samples shared the same dominant SBS signature, though the authors noted the sample mutation calls were highly concordant in covered exonic regions between the two data types (Bailey et al. 2020). Thus, while WES and WGS can yield consistent signature analysis results, WGS provides a more comprehensive assessment of the mutational landscape by enabling detection of mutations in non‐coding regions, structural variation, and other genomic alterations. Moreover, WES is known to be more susceptible to sequencing errors such as PCR bias and variability in coverage due to GC‐rich regions, which can affect signature assignment and attributions. The lower number of mutations captured by WES can also reduce statistical power to detect signatures, especially those with relatively “flat profiles” (Abbasi and Alexandrov 2021). However, given the absence of a bulk mutational effect, together with prior studies that utilized clonal‐based WGS approaches in models of short‐term arsenic exposure and similarly failed to identify an arsenic‐specific mutational signature (Kucab et al. 2019; Speer, Nandi, et al. 2023), we consider that applying a similar strategy to our cellular models of chronic arsenic damage (better representing real‐world exposures) to reveal an arsenic‐specific signature may be limited. Nevertheless, WGS remains an avenue for further investigation, as structural variation has been largely understudied in prior studies and may be more sensitive to arsenic‐related effects.

In conclusion, leveraging tumor tissue‐derived WES data and toenail arsenic measurements from 17 patients in Bangladesh along with two complementary in vitro models of chronic arsenic exposure, we found that arsenic is not clearly linked to a reproducible pattern of somatic mutations. Our study builds upon prior work utilizing sequencing approaches to assess arsenic's mutational impact, including one study of a single arsenic‐exposed lung cancer patient (Martinez et al. 2013), and two experimental studies of short arsenic exposure (Speer, Nandi, et al. 2023; Kucab et al. 2019). Consistent with these studies, our results suggest arsenic may not act as an independent mutagen or give rise to a consistent mutational signature. Instead, one possible explanation is that arsenic may contribute to cancer through co‐carcinogenic mechanisms involving diverse cellular and epigenetic pathways without producing a reproducible mutational signature. However, given the limitations of our study, future studies with larger sample sizes, greater sensitivity for detecting low‐frequency mutations, and a WGS‐based approach are needed to further investigate the role of arsenic in carcinogenesis.

## Author Contributions

N.J. contributed to study conception, performed analyses, interpreted the data, and wrote the main manuscript text. K.D. contributed to study design and implementation. E.W. helped implement the somatic mutation calling pipeline. M.G.K. and F.J. generated whole‐exome sequencing data and provided technical guidance on the somatic mutation calls. S.E.H., M.K., S.H., Q.C., F.A., and H.A. coordinated patient recruitment and sample collection. E.W. and Y.‐Y.H. provided cell line samples and contributed to data interpretation. L.Y. provided guidance on project direction and data interpretation. B.L.P. conceived the project and contributed to writing, editing, and data interpretation.

## Funding

This study was supported by National Institute of Environmental Health Sciences R35 ES028379 (to B.L.P.), National Institute of Environmental Health Sciences P30 ES027792 (to H.A.), and National Institute of Environmental Health Sciences R35 ES031693 (to Y.Y.H.).

## Ethics Statement

The study received institutional review board (IRB) clearance from Bangladesh Medical Research Council (BMRC) and The University of Chicago.

## Consent

Written informed consent was obtained from all study participants. Participants received no compensation.

## Conflicts of Interest

The authors declare no conflicts of interest.

## Supporting information


**Table S1:** ANNOVAR annotation of functional mutations in known driver genes in bladder urothelial cancer samples (*n* = 9).
**Table S2:** Number of functional mutations in known driver genes in bladder urothelial cancer samples (*n* = 9).

**Figure S1:** Distribution of median coverage for whole‐exome sequenced tumor samples (*n* = 66). Vertical line denotes 30× coverage.
**Figure S2:** Distribution of arsenic exposure across residential districts represented in our Bangladesh patient cohort (*n* = 51). District‐level exposure levels is defined as the proportion of tube wells with arsenic (As) concentrations greater than 10 μg/L, based on data from Chakraborti et al. 2010. Counts represent the number of patients from each district.
**Figure S3:** Element concentrations in human toenails (*n* = 17).
**Figure S4:** Mutational burden and mutational signature patterns after applying minimum variant allele frequency (5%) and read depth (25×) thresholds (A) Toenail arsenic concentrations per sample. (B) Number of single base substitutions (SBS) per megabase (Mb) per sample. (C) Number of mutations attributed to each COSMIC mutational signature. Mutational signatures contributing less than 10% of mutations to the total mutational profile are not shown. For each cancer subtype, the ordering of bars in panels B, C is based on sample arsenic concentrations shown in panel A. pRCC, papillary renal cell carcinoma; SCLC, small cell lung carcinoma; sRCC, sarcomatoid renal cell carcinoma.
**Figure S5:** Proportion of mutations excluded for each SBS type after applying minimum variant allele frequency (5%) and read depth (25×) thresholds. Paired Wilcoxon signed‐rank tests were used to compare the proportion of C>T substitutions filtered with that of each other SBS type.
**Figure S6:** Overall elemental burden does not reveal any noticeable patterns with mutational burden or signature profiles (A) Toenail element concentrations per sample. Concentrations normalized across individuals using z‐scores (B) Number of single base substitutions (SBS) per megabase (Mb) per sample. (C) Number of small insertions and deletions (indel) per Mb per sample. (D) Number of mutations attributed to each COSMIC SBS mutational signature. (E) Number of mutations attributed to each COSMIC indel mutational signature. Mutational signatures contributing less than 10% of mutations to the total mutational profile are not shown. For each cancer subtype, the ordering of bars in panels is based on sample SBS in panel B. pRCC, papillary renal cell carcinoma; SCLC, small cell lung carcinoma; sRCC, sarcomatoid renal cell carcinoma. The elements measured included aluminum (Al), vanadium (V), chromium (Cr), iron (Fe), manganese (Mn), cobalt (Co), nickel (Ni), copper (Cu), zinc (Zn), molybdenum (Mo), cadmium (Cd), tin (Sn), lead (Pb), arsenic (As), selenium (Se), antimony (Sb).
**Figure S7:** Comparison of total mutation burden and mutations attributed to signatures in arsenic‐exposed versus non‐treated HaCaT skin epithelial cells. (A) Single base substitutions (SBS), (B) double base substitutions (DBS), and (C) small insertions and deletions (indel). NT: non‐treated cells; As‐treated: arsenic‐treated cells.
**Figure S8:** Comparison of total mutation burden and mutations attributed to signatures in arsenic‐exposed versus non‐treated BEAS‐2B lung epithelial cells. (A) Single base substitutions (SBS) and (B) small insertions and deletions (indel). NT: non‐treated cells; As‐treated: arsenic‐treated cells.

## Data Availability

Individual‐level genetic data and associated metadata will be made available on dbGaP (Arsenic Exposure Mutation Signature, phs004934.v1.p1).
