# Supplementary material for: Assessing the Mutational Consequences of Chronic Arsenic Exposure Using Patient‐Derived Tumor Samples and In Vitro Systems
Source: Environ Mol Mutagen. 2026 Oct 24;67(8-9):e70079. doi: 10.1002/em.70079 (PMC13695014; doi:10.1002/em.70079)
Supplement: Supplementary file 1 [file EM-67-0-s001.pdf]

Supplementary Figures

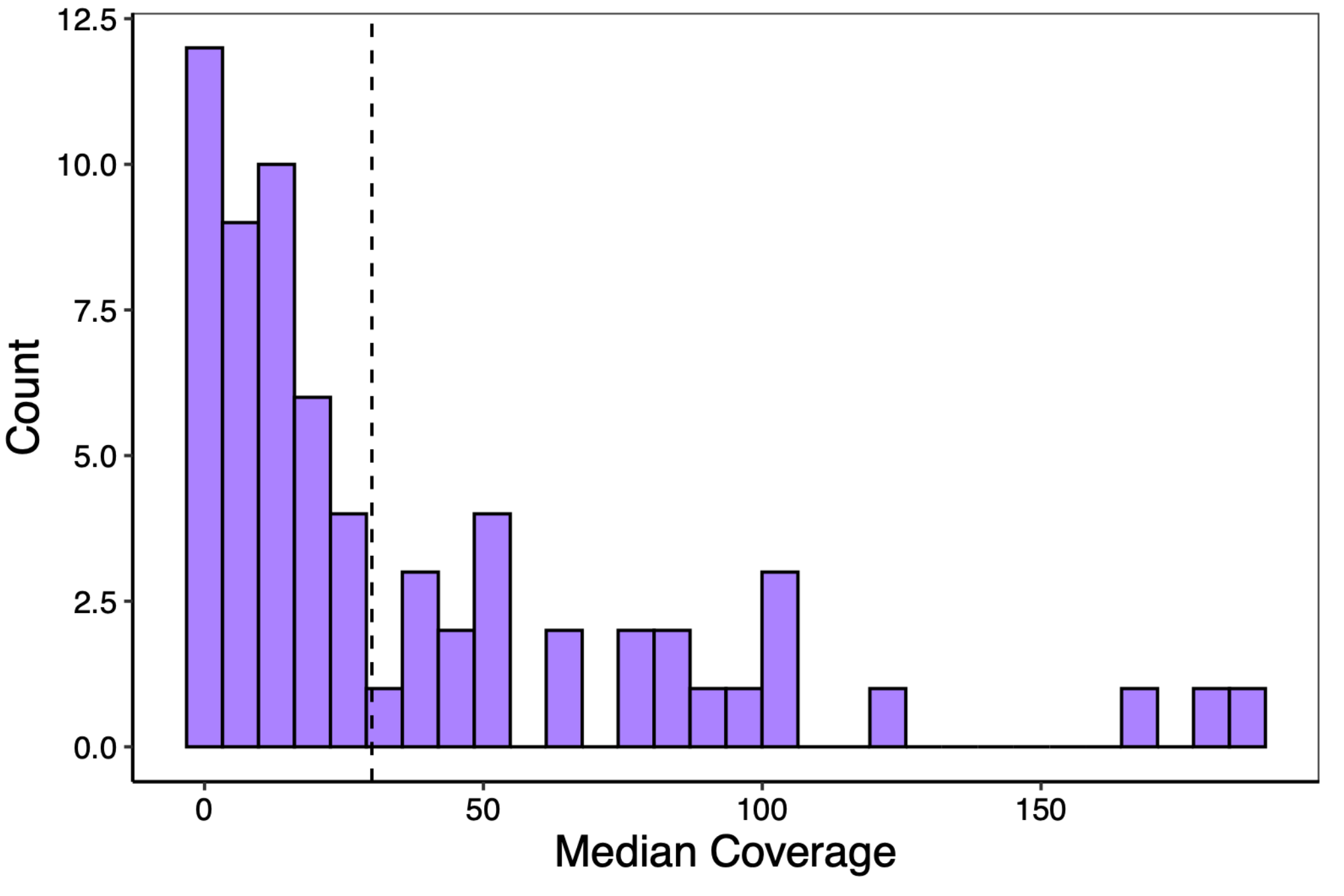

Figure S1. Distribution of median coverage for whole-exome sequenced tumor samples (n=66). Vertical line denotes 30x coverage.

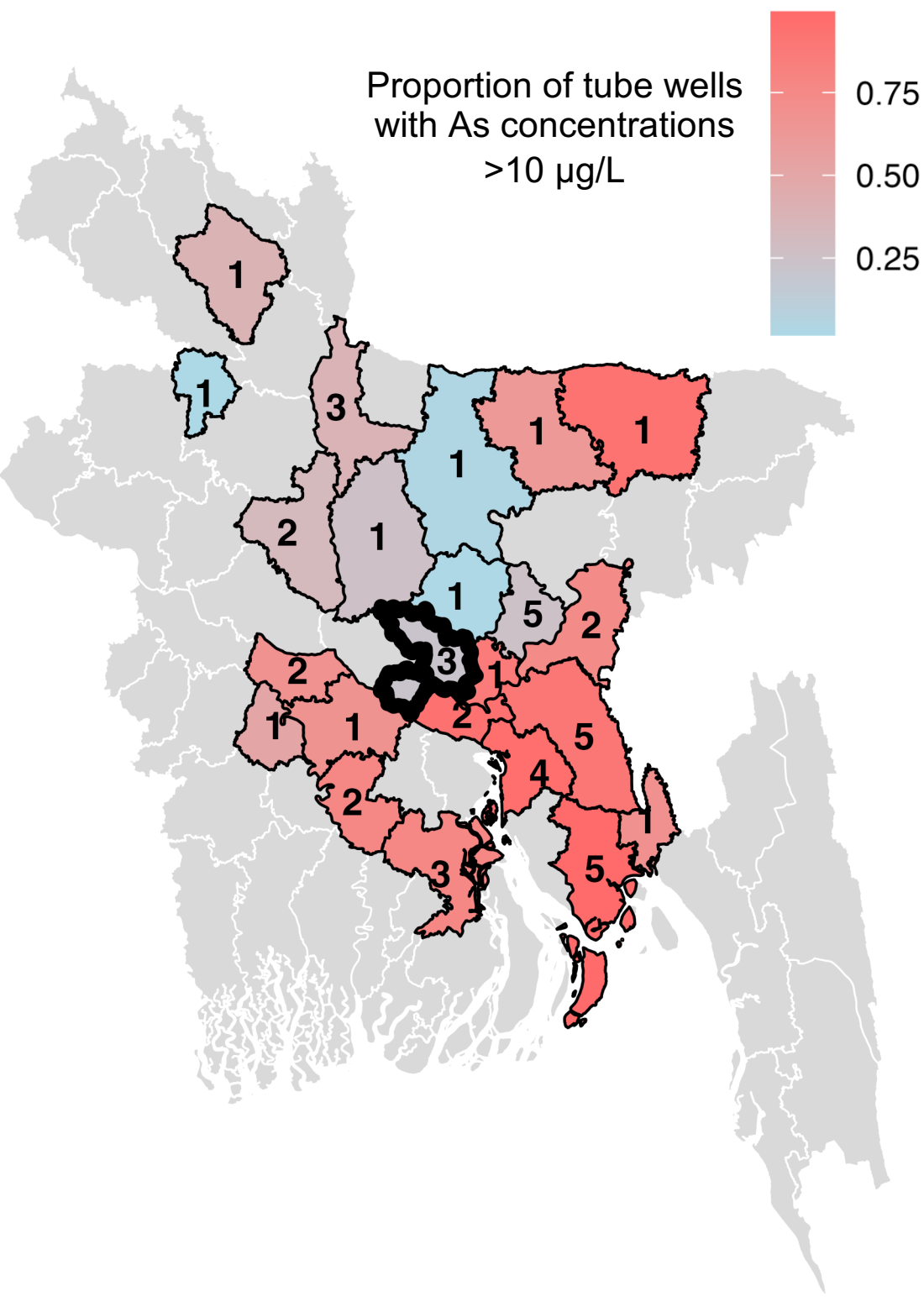

Figure S2. Distribution of arsenic exposure across residential districts represented in our Bangladesh patient cohort (n=51). District-level exposure levels is defined as proportion of tube wells with arsenic (As) concentrations greater than 10 µg/L, based on data from Chakraborti et al., 2010. Counts represent the number of patients from each district.

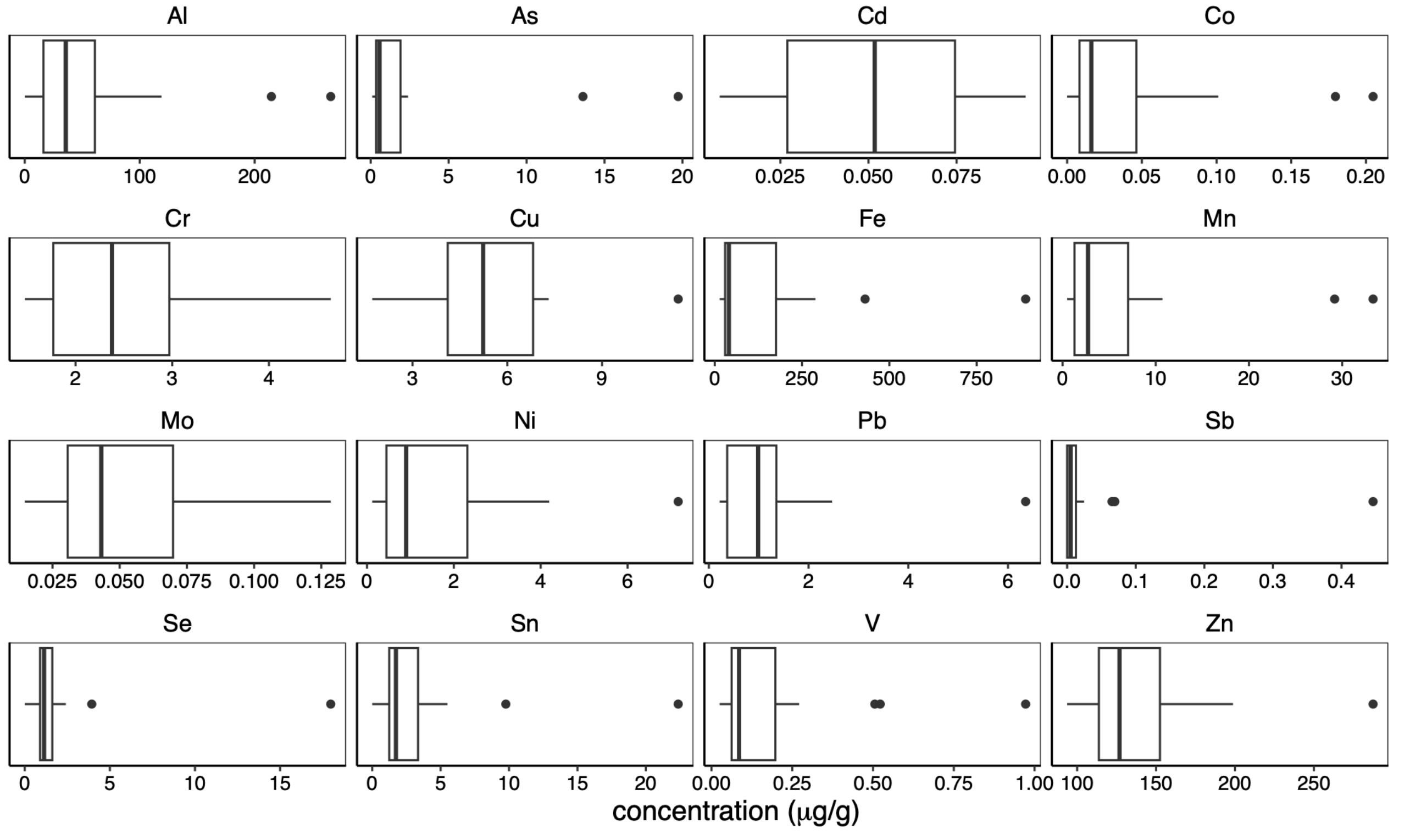

Figure S3. Element concentrations in human toenails (n=17)

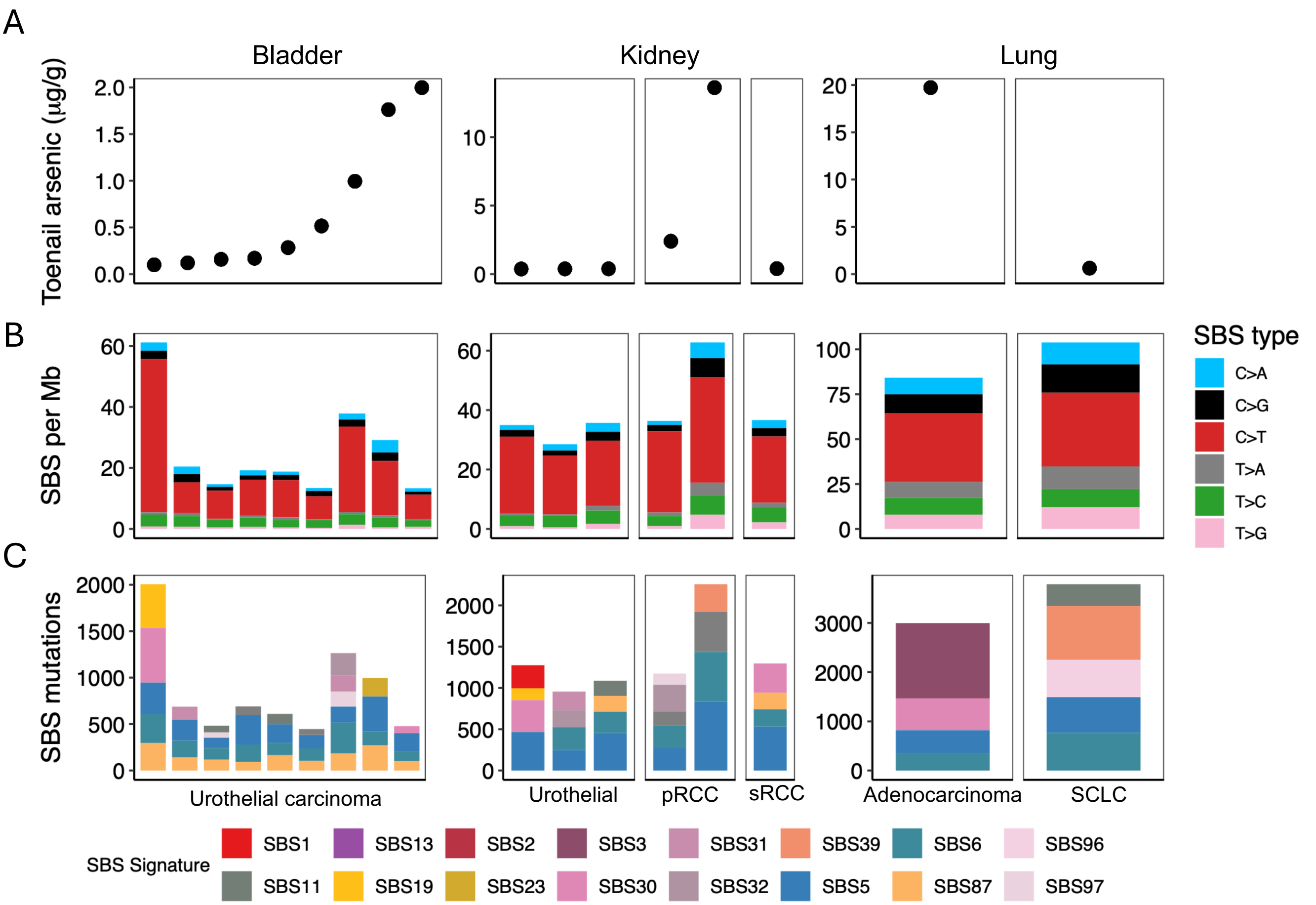

Figure S4. Mutational burden and mutational signature patterns after applying minimum variant allele frequency (5%) and read depth (25x) thresholds (A) Toenail arsenic concentrations per sample. (B) Number of single base substitutions (SBS) per megabase (Mb) per sample. (C) Number of mutations attributed to each COSMIC mutational signature. Mutational signatures contributing less than 10% of mutations to the total mutational profile are not shown. For each cancer subtype, the ordering of bars in panels B-C is based on sample arsenic concentrations shown in panel A. pRCC: papillary renal cell carcinoma; sRCC: sarcomatoid renal cell carcinoma; SCLC: small cell lung carcinoma

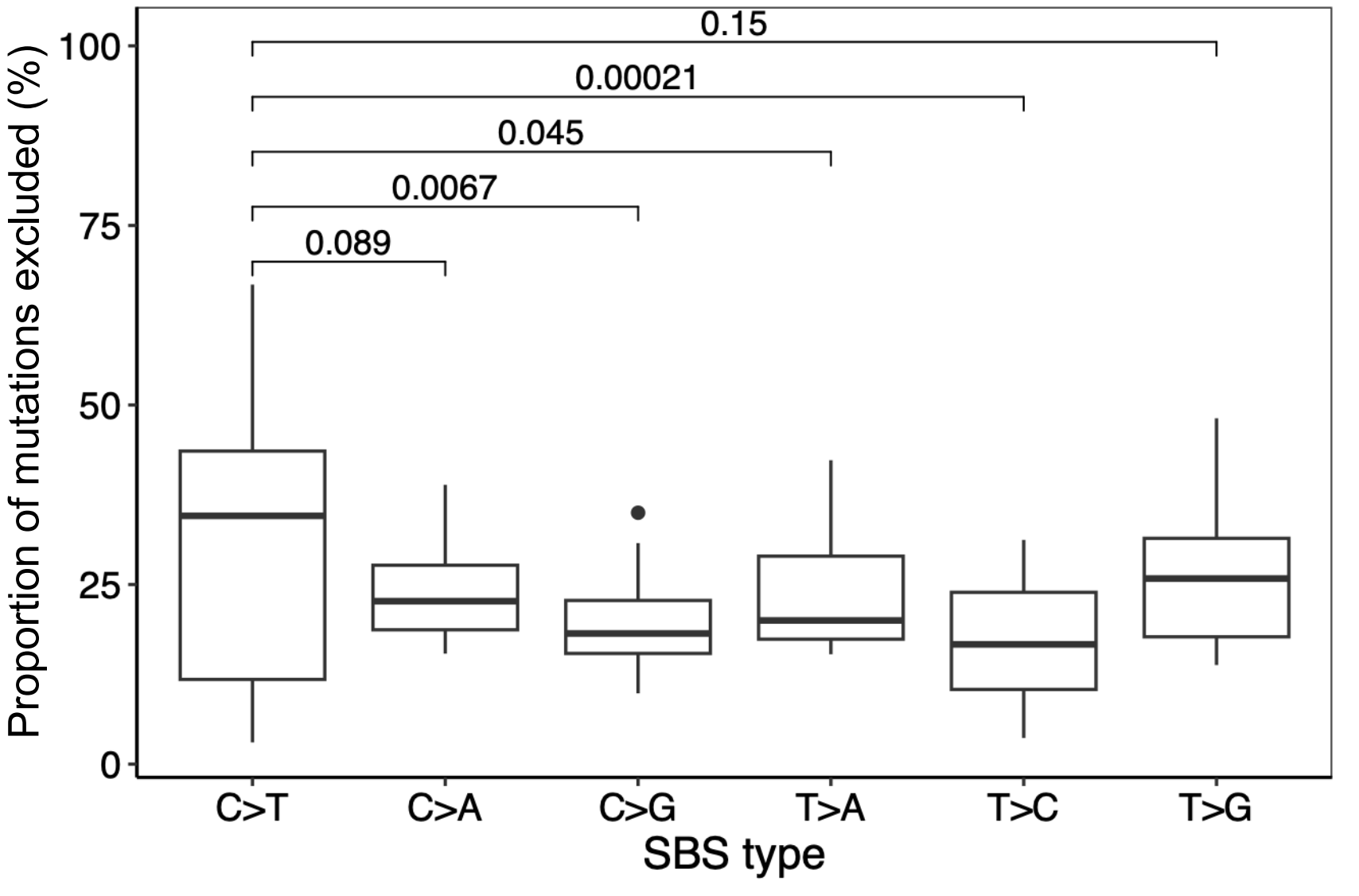

Figure S5. Proportion of mutations excluded for each SBS type after applying minimum variant allele frequency (5%) and read depth (25x) thresholds. Paired Wilcoxon signed-rank tests were used to compare the proportion of C>T substitutions filtered with that of each other SBS type.

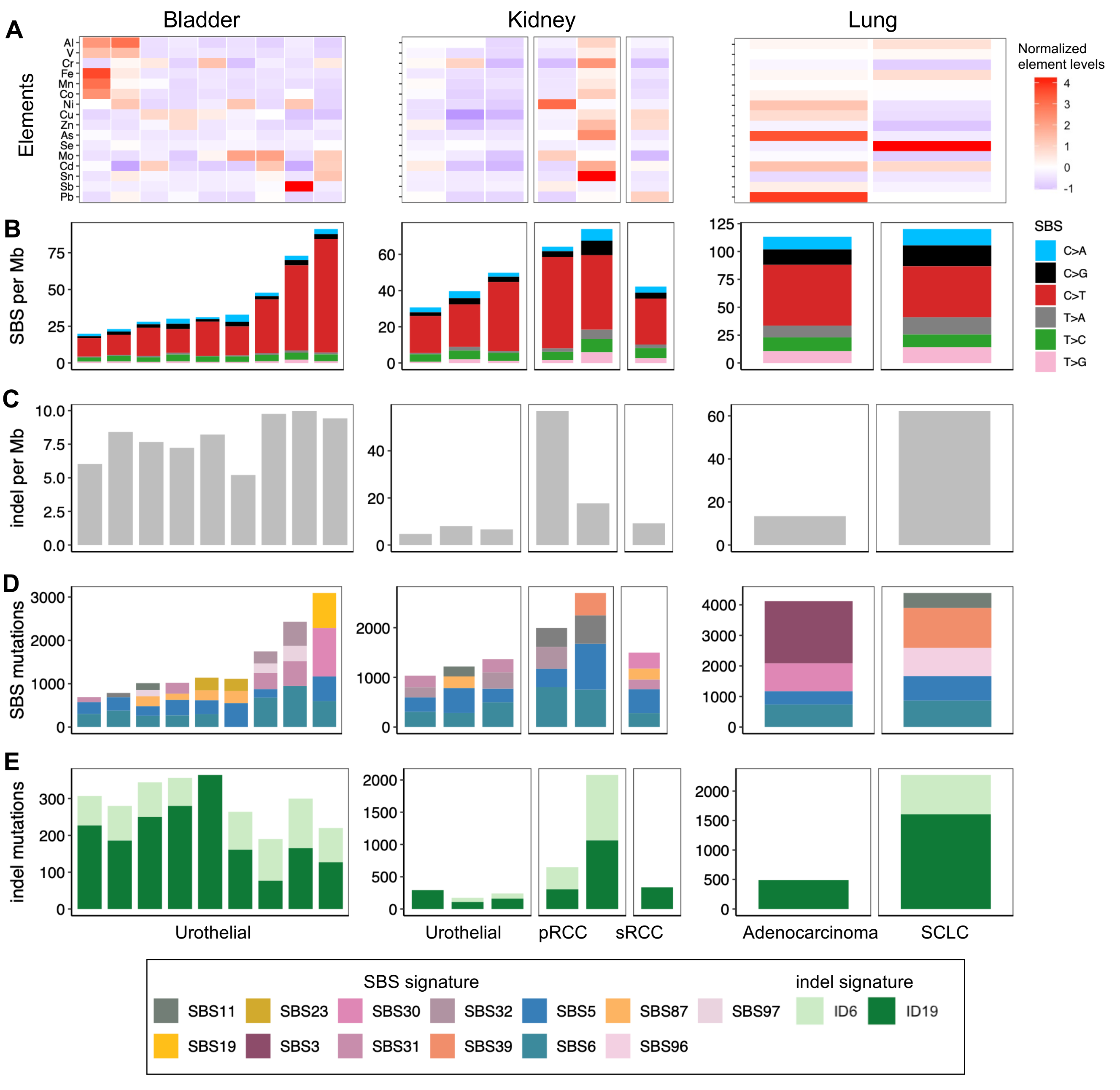

Figure S6. Overall elemental burden does not reveal any noticeable patterns with mutational burden or signature profiles (A) Toenail element concentrations per sample. Concentrations normalized across individuals using z scores (B) Number of single base substitutions (SBS) per megabase (Mb) per sample. (C) Number of small insertions and deletions (indel) per Mb per sample. (D) Number of mutations attributed to each COSMIC SBS mutational signature. (E) Number of mutations attributed to each COSMIC indel mutational signature. Mutational signatures contributing less than 10% of mutations to the total mutational profile are not shown. For each cancer subtype, the ordering of bars in panels is based on sample SBS in panel B. pRCC: papillary renal cell carcinoma ; sRCC: sarcomatoid renal cell carcinoma; SCLC: small cell lung carcinoma. The elements measured included aluminum (Al), vanadium (V), chromium (Cr), iron (Fe), manganese (Mn), cobalt (Co), nickel (Ni), copper (Cu), zinc (Zn), molybdenum (Mo), cadmium (Cd), tin (Sn), lead (Pb), arsenic (As), selenium (Se), antimony (Sb).

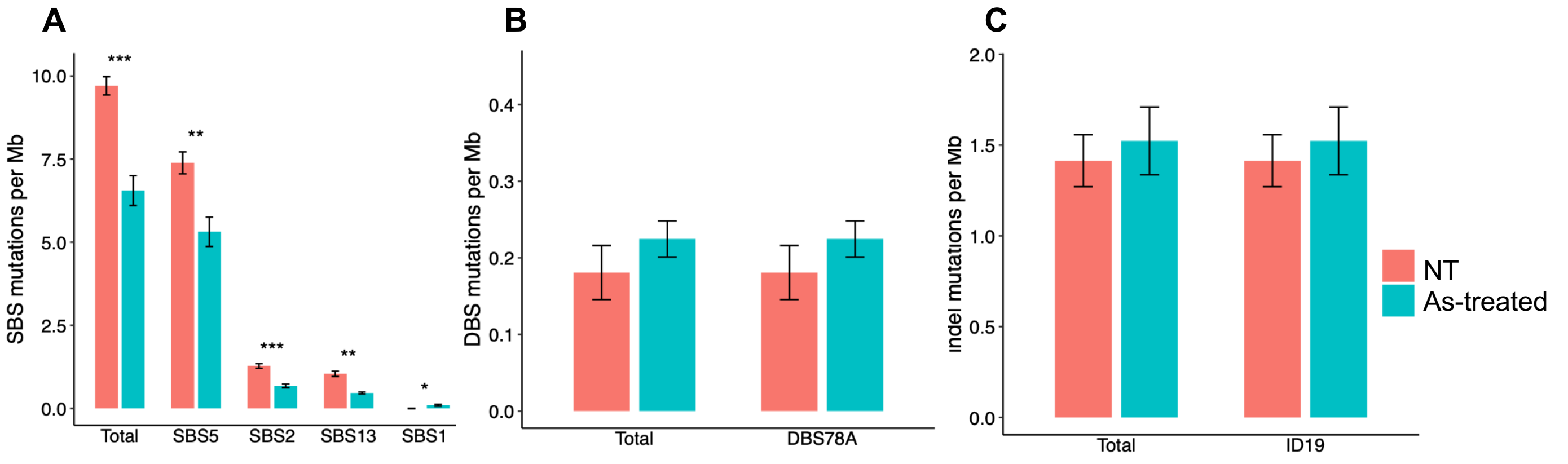

Figure S7. Comparison of total mutation burden and mutations attributed to signatures in arsenic-exposed versus non-treated HaCaT skin epithelial cells. (A) Single base substitutions (SBS), (B) double base substitutions (DBS), and (C) small insertions and deletions (indel). NT: non-treated cells; As-treated: arsenic treated cells

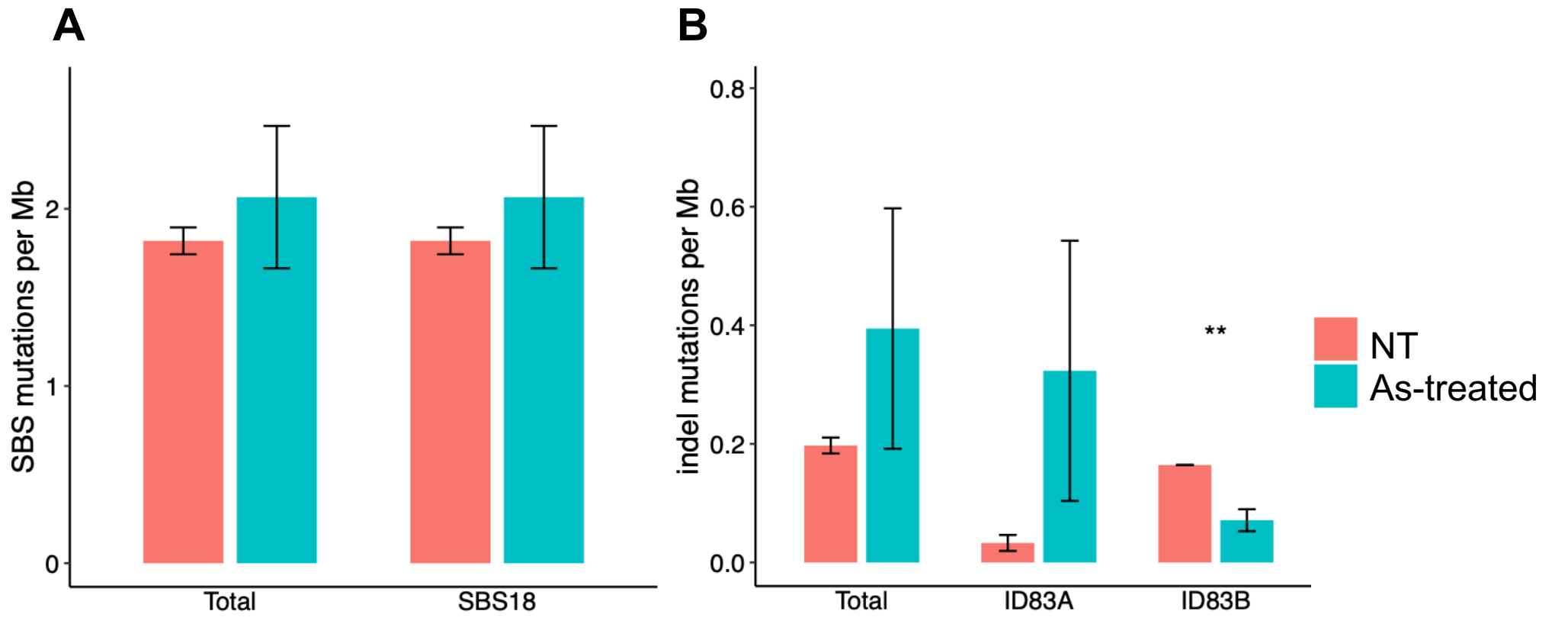

Figure S8. Comparison of total mutation burden and mutations attributed to signatures in arsenic-exposed versus non-treated BEAS-2B lung epithelial cells. (A) Single base substitutions (SBS) and (B) small insertions and deletions (indel). NT: non-treated cells; As-treated: arsenic treated cells
